# *Trigonella foenum-graecum* L. and *Psoralea corylifolia* L. Improve Erectile Dysfunction in Streptozotocin-Induced Diabetic Rats through Suppression of Oxidative Stress

**DOI:** 10.1155/2022/4187359

**Published:** 2022-06-06

**Authors:** Shujun Jiang, Quanyan Cai, Yang Gao, Hui Dong, Yan Zhao, Fan Wu, Xin Ba, Yang Luan, Xin Zou, Lijun Xu, Fuer Lu

**Affiliations:** ^1^Department of Integrated Traditional Chinese and Western Medicine, Tongji Hospital, Tongji Medical College, Huazhong University of Science and Technology, Wuhan 430030, China; ^2^Institute of Integrated Traditional Chinese and Western Medicine, Tongji Hospital, Tongji Medical College, Huazhong University of Science and Technology, Wuhan 430030, China; ^3^Beijing Tcmages Pharmaceutical Co., Ltd., Beijing 101301, China; ^4^Department of Urology, Tongji Hospital, Tongji Medical College, Huazhong University of Science and Technology, Wuhan 430030, China

## Abstract

**Background:**

Diabetes mellitus-induced erectile dysfunction (DMED) is one of the most common complications of diabetes and is mainly attributed to oxidative stress. Hu-Lu-Ba-Wan (HLBW) is a classic Chinese formulation consisting of *Trigonella foenum-graecum* L. (TFG) and *Psoralea corylifolia* L. (PC). HLBW has been used not only for the treatment of diabetes but also for the treatment of erectile dysfunction in clinics. This study aimed to explore the efficacy and underlying mechanism of HLBW in ameliorating erectile function in streptozotocin-induced diabetic rats.

**Methods:**

The diabetic model was established by tail vein injection of streptozotocin (26 mg/kg), and then DMED rats screened by the apomorphine test were randomly divided into two groups: the model group and the HLBW group. The rats in the HLBW group were administered HLBW granules daily for 12 weeks. Fasting blood glucose and fasting insulin were tested by a commercial kit. Intracavernous pressure (ICP) and mean arterial pressure (MAP) were measured by cavernous nerve electrostimulation before the rats were killed. Erectile function was evaluated with ICP/MAP. The markers of oxidative stress in the corpus cavernosum (CC) were assayed by assay kits. Apoptosis in cavernosal tissue was detected by Western blotting (WB). The expression levels of vascular endothelial marker (vWF), *α*-smooth muscle actin (*α*-SMA), endothelial nitric oxide synthase (eNOS), and NADPH oxidase subunit P47^phox^ were determined by WB and PCR. Furthermore, the structure of the CC was further confirmed by Masson's trichrome staining.

**Results:**

The results showed that HLBW significantly reduced blood glucose and increased insulin sensitivity. HLBW reduced oxidative stress and apoptosis. In addition, we observed that the expression levels of vWF, *α*-SMA, and eNOS as well as the ratio of smooth muscle to collagen increased in the HLBW group.

**Conclusions:**

Our results demonstrated that HLBW could reduce oxidative stress damage in CC to improve diabetes mellitus-induced erectile dysfunction in rats by inhibiting NADPH oxidase.

## 1. Introduction

With the development of the social economy and changes in people's lifestyles, the prevalence of type 2 diabetes is increasing. According to the latest survey from the International Diabetes Federation (IDF), diabetes mellitus (DM) affects more than 450 million people globally, and the figure is expected to rise to 693 million by 2045 [1]. DM has been widely considered one of the major risk factors for the development of erectile dysfunction (ED). The incidence of ED in diabetic patients is threefold higher than that in the general population [2, 3]. Since the 1970s, the association between diabetes and the development of ED has been confirmed in clinical trials [4]. DM-induced ED (DMED) has attracted increasing attention worldwide. Some studies have shown that DMED is the result of multiple pathological mechanisms, including the accumulation of glycation end products, increased levels of oxygen free radicals, endothelial dysfunction, neuropathic damage, and penile fibrosis [3, 5, 6]. Phosphodiesterase type 5 (PDE5) inhibitors have made a major breakthrough as a first-line drug for treating ED. However, patients with DMED show a worse response to these drugs than ED patients without diabetes [7]. Previous studies indicated that the treatment of DMED on the basis of controlling blood sugar was superior to monotherapy with PDE5 inhibitors [8, 9]. Therefore, it is necessary to find a drug that can not only control glucose but also treat ED.

Substantial studies have demonstrated that oxidative stress plays an important role in the occurrence of DMED [10]. DM is a metabolic disease characterized by hyperglycemia, and persistently high blood sugar can increase reactive oxygen species (ROS) production. The generation of ROS leads to endothelial dysfunction and reduces the bioavailability of nitric oxide (NO), which impairs the relaxation function of cavernosal smooth muscle [11]. Nicotinamide adenine dinucleotide phosphate (NADPH) oxidases are major sources of ROS production. Increasing evidence has suggested that NADPH oxidase-derived ROS production plays a potent role in initiating and accelerating the development of DMED [12]. NADPH oxidase inhibitors can ameliorate DMED by reducing ROS production [12, 13].

Herbal medicines are well recognized to treat diabetes and its complications by the public in China. Hu-Lu-Ba-Wan (HLBW), composed of *Trigonella foenum-graecum* L. (TFG) and *Psoralea corylifolia* L. (PC), is a classical herbal prescription used to treat Shen (kidney)-yang deficiency in traditional Chinese medicine. TFG, which has the characteristics of tonifying kidney, eliminating cold, and relieving pain, is mainly used for kidney-yang deficiency, impotence, and spermatorrhea. PC, which can warm kidney to help Yang and warm the spleen to stop diarrhea, is mainly used for impotence, spermatorrhea, and diarrhea in the morning. The phytochemical studies revealed that TFG contained steroids, alkaloids, saponins, polyphenols, flavonoids, lipids, carbohydrates, amino acids, and hydrocarbons, and PC contained flavonoids, coumarins, phenols, benzofurans, benzopyrans, quinines, sesquiterpenoids, triterpenoids, and steroids. TFG and PC are often used to treat diabetes and sexual dysfunction in the clinic. TFG mainly consists of trigonelline and dioscin, which has been proved to reduce glucose and increases the activity of antioxidation [14]. PC is mainly composed of psoralen, isopsoralen, and corylifolinin, which can ameliorate diabetes by inhibiting oxidative stress [15–17]. Our previous research demonstrated that HLBW can improve oxidative stress in the testes of diabetic rats via the PKC*α*/NAPDH oxidase signaling pathway [18]. Therefore, the aim of this study was to determine whether HLBW can inhibit oxidative stress to ameliorate DMED by downregulating NADPH oxidase.

## 2. Materials and Methods

### 2.1. Chemicals and Reagents

Streptozotocin (STZ) was purchased from Merck & Co. Inc. (Regierungsbezirk Darmstadt, Hesse-Damstadt, Germany). The amplification primers were provided by GenScript Biotechnology Co. Ltd. (Nanjing, China). RNA extraction kits, reverse transcription kits, and amplification kits were obtained from Yeasen Biotech Co. Ltd. (Shanghai, China). Superoxide dismutase (SOD), malondialdehyde (MDA), and the radioimmunoassay kits for insulin were purchased from Jiancheng Bioengineering Institute (Nanjing, China). Bicinchoninic acid (BCA) protein assay kits, RIPA lysis buffer, protease inhibitor cocktail, and a kit for sodium dodecyl sulfate-polyacrylamide gel electrophoresis were obtained from Wuhan Servicebio Technology Co. Ltd. (Wuhan, China). Hematoxylin-eosin was purchased from Wuhan Servicebio Technology Co. Ltd. (Wuhan, China). Rabbit anti-p47^phox^(p47) (sc-17845), anti-(Bcl-2) (sc-7382), and anti- (vWF) (sc-365712) were purchased from Santa Cruz Biotechnology. Rat anti-*α*-SMA (19245S), anti-Bax (2772S), anti-eNOS (4231S), anti-rabbit IgG (5151P), anti-rat IgG (5257P), and horseradish peroxidase-linked antibodies were purchased from Cell Signaling Technology, Inc. (Danvers, MA, USA).

### 2.2. HLBW Preparation

The two Chinese herbs in HLBW, which is composed of TFG and PC concentrated granules extracted from preparations of traditional Chinese medicine, were purchased from Kangrentang Pharmaceutical Co. Ltd. (Beijing, China). According to modern extraction technology, each gram of TFG granules is equivalent to 10 g decoction pieces, while each gram of PC granules is equivalent to 20 g decoction pieces. The therapeutic dosage of HLBW in rats is six times the dosage in humans per kilogram. TGF and PC were mixed in a ratio of 1:1 according to the dosage of decoction pieces; thus, 1.5 g HLBW concentrated granules (containing 0.5 g PC concentrated granules and 1 g TFG concentrated granules) were dissolved in 2.5 mL water (0.6 g/mL).

### 2.3. High-Performance Liquid Chromatography (HPLC) Analysis of HLBW

HPLC was used to analyze the chemical composition of HLBW. Chromatographic separation was carried out on an ACQUITY UPLC BEH C_18_ column (100 mm ^*∗*^2.1 mm, 1.7 *μ*m). The mobile phase was composed of acetonitrile (A) and 0.1% phosphoric acid (B) at a flow rate of 0.35 mL/min with a gradient eluting detailed system. About 1 *μ*L of the sample was added to the instrument and examined at a 248 nm detection wavelength.

### 2.4. Animal Grouping and Drug Treatment

A total of 34 specific pathogen-free (SPF) male Wistar rats weighing 180–210 g were purchased from Hubei Experimental Animal Center (Wuhan, China; Certificate NO. 42000600026939). The rats were kept in the SPF room of the Experimental Animal Center of Huazhong University of Science and Technology. All animals had free access to food and water under controlled temperature (22 ± 1°C) and humidity (45%–55%) and a 12 h light-dark cycle. All studies were conducted in accordance with the National Institutes of Health (NIH) publication and Huazhong University of Science and Technology principles for laboratory animal use and care. A total of eight rats were randomly divided into the control group and fed a standard diet, while the others (*n* = 26) were fed a high-fat diet (67.5% standard laboratory rat chow, 15% lard, 15% sugar, 2% cholesterol, and 0.5% bile salts) for 4 weeks. The rats fed a high-fat diet were injected with STZ at a dose of 26 mg/kg via the caudal vein after overnight fasting [18]. One week later, the rats with a random blood glucose level over 16.7 mmol/L were identified as diabetic. A total of 22 rats with DM survived. After 8 weeks of high-fat feeding, an apomorphine (APO) solution was subcutaneously injected into the necks of rats at 100 *μ*g/kg. Then, the number of erections in the rats was observed and recorded for 30 min, and a total score of 0 was considered DMED (*n* = 16). The DMED rats were randomly assigned to the nonintervention group (model group; *n* = 8) and HLBW-treated group (HLBW group; *n* = 8). The rats in the HLBW group were treated with HLBW concentrated granules at a dose of 1.35 g/kg (equivalent to 18 g/kg HLBW decoction pieces) by gavage once a day for 12 weeks [19]. After overnight fasting, all rats were anesthetized with 0.4% sodium pentobarbital (50 mg/kg) by intraperitoneal injection. First, erectile function is measured, and then blood samples were collected from the abdominal aorta. The serum was separated by centrifuging at 3,000 rpm for 15 min at 4°C and stored at −80°C in aliquots until examined. After taking blood samples, the rats were sacrificed by cervical dislocation. Then, excess tissue was quickly removed from the surface of penile tissue and the corpus cavernosum (CC) was divided into three parts. The middle part was sliced after being fixed and embedded with paraffin, and the remaining parts were stored in a freezer at −80°C.

### 2.5. Evaluation of Erectile Function

A 25-gauge infusion needle filled with heparin solution and connected to a polyethylene (PE)-50 tube was inserted longitudinally into the penile crus, and the cavernous nerve was electrically stimulated at 5 V, 2 mA, 15 Hz, and a pulse width of 1.2 ms for 1 min to record the intracavernous pressure (ICP). A PE-50 tube, which was rinsed in advance with heparin sodium, was carefully inserted into the left carotid artery to measure the mean arterial pressure (MAP) under the same conditions. The intervals of the two electrical stimulations were 5 min. The ICP and MAP data were collected by a multichannel biosignal acquisition and analysis system. The ratio of peak ICP and area under the curve (AUC) to MAP were calculated.

### 2.6. Measurement of Fasting Blood Glucose (FBG) and Fasting Insulin (FINS)

FBG levels were examined with the glucose oxidase method using a semiautomatic biochemical analyzer, whereas FINS levels were tested using radioimmunoassay kits. The indicator of insulin resistance (IR) was evaluated using homeostatic model assessment (HOMA), and the calculation method of HOMA-IR was (FBG × FINS)/22.5.

### 2.7. Analysis of Oxidative Stress Indices in the Corpus Cavernosum (CC)

The oxidative stress markers in the CC were tested as follows: frozen corpus cavernosum tissue (50 mg/rat) was homogenized in 1 mL normal saline. The homogenate was centrifuged at 3,000 rpm for 15 min at 4°C, and the supernatant was collected. The protein concentrations were measured using the BCA method. Concentrations of MDA in the cavernous body were measured with thiobarbituric acid (TBA) detection, and SOD activity was measured with xanthine oxidase (XOD) detection under the guidance of the manufacturer's instructions.

### 2.8. Masson's Trichrome Staining

Paraffin-embedded samples were sectioned to a thickness of 5 *μ*m. After dewaxing, the sections were prepared for Masson's trichrome staining that shows smooth muscle in red areas and collagen fiber in blue areas in the CC. The numbers of smooth muscle fibers and collagen fibers were observed under light microscopy, and the ratio was calculated using ImageJ software.

### 2.9. Western Blot Analysis

Total proteins were extracted from frozen corpus cavernosum tissue, and the concentrations were measured by a BCA assay kit. Protein samples from each sample were separated by 10% sodium dodecyl sulfate-polyacrylamide gel electrophoresis (SDS-PAGE) and then transferred to nitrocellulose membranes. Next, the membranes were blocked with 5% fat-free milk dissolved in phosphate-buffered saline-Tween (PBST) for 2 h at room temperature, and the target protein was incubated overnight with primary antibodies (P47^phox^, Bax, Bcl-2, *α*-SMA, eNOS, and vWF) at 4°C. After rinsing with PBST 3 times, the membranes were incubated with the fluorescent secondary antibody for 1 h. Finally, the expression of target proteins was visualized using Odyssey Infrared Imaging and analyzed with ImageJ software under GAPDH as a unified standard.

### 2.10. Quantitative Real-Time PCR Analysis

Total RNA was extracted from frozen corpus cavernosum tissue using TRIzol reagent. Reverse transcription was performed using the extracted RNA with a PrimeScript RT reagent kit at 37°C for 15 min and 85°C for 5 s on a Mastercycler gradient PCR instrument (Eppendorf Company, Hamburg, Germany). The prepared cDNA was amplified by a real-time PCR system under definite thermal conditions. The mRNA expression levels of *α*-SMA, eNOS, vWF, and P47^phox^ were detected according to the fluorescence signal. The data were calculated using the 2^−△△CT^ method with GAPDH as the standardized control. Primer sequences of the target genes are shown in Table 1.

### 2.11. Statistical Analysis

The data were statistically analyzed by SPSS 22.0 software and expressed as the mean ± standard deviation ($\bar{x}$ ± SD). One-way analysis of variance (ANOVA) and Dunnett's *t*-test were used to analyze the differences among groups, and *P* < 0.05 was considered to indicate significant differences.

## 3. Results

### 3.1. HPLC Fingerprinting of Major Components of HLBW Granules

**Figure 1:**
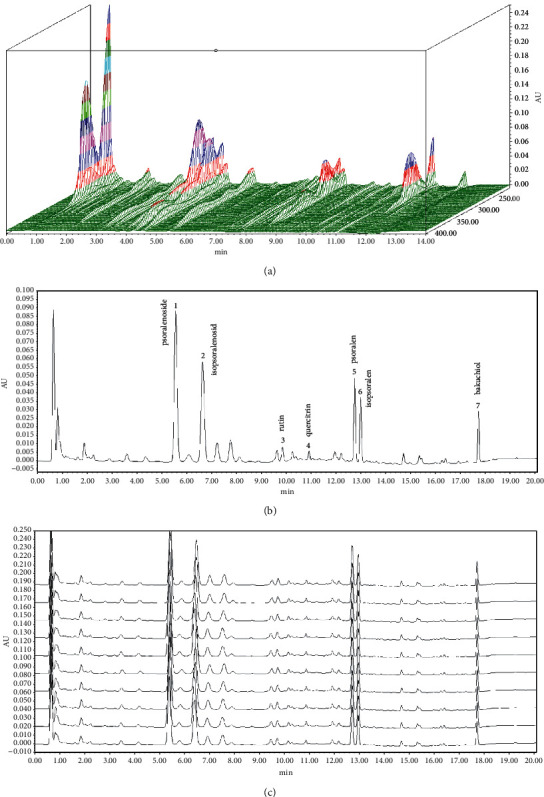
HPLC fingerprinting chromatogram of HLBW granules. (a) 3D-HPLC fingerprint of HLBW. (b) HPLC chromatograms of seven reference standards. (c) HPLC chromatograms of 10 different HLBW batches. In the chromatograms, (1) psoralenoside; (2) isopsoralenosid; (3) rutin; (4) quercitrin; (5) psoralen; (6) isopsoralen; and (7) bakuchiol.

The 3D-HPLC chromatogram of HLBW granules is shown in Figure 1(a). Seven main constituents of HLBW were identified by comparing the retention time and peak height with the reference standard. The HPLC fingerprint chromatogram of reference standards is shown in Figure 1(b), and HPLC fingerprint chromatograms of the HLBW extracts in 10 different batches are shown in Figure 1(c). Ten batches of HLBW samples were examined, and the HPLC chromatogram had similarity coefficients greater than 0.99.

### 3.2. Effect of HLBW on Body Weight, FBG, FINS, and HOMA-IR in DMED Rats

The results of body weight, FBG, FINS, and HOMA-IR among the three groups are shown in Table 2. The body weight was significantly lower in the DMED group than in the control group (*P* < 0.001), while HLBW treatment increased the body weight (*P* < 0.05). The levels of FBG, FINS, and HOMA-IR were obviously elevated in the DMED group compared with the control group (*P* < 0.001), and HLBW administration significantly increased the levels of FBG, FINS, and HOMA-IR (*P* < 0.01). The results indicated that HLBW treatment could improve glucose tolerance and enhance insulin sensitivity.

### 3.3. HLBW Treatment Improves Erectile Dysfunction in DMED Rats

**Figure 2:**
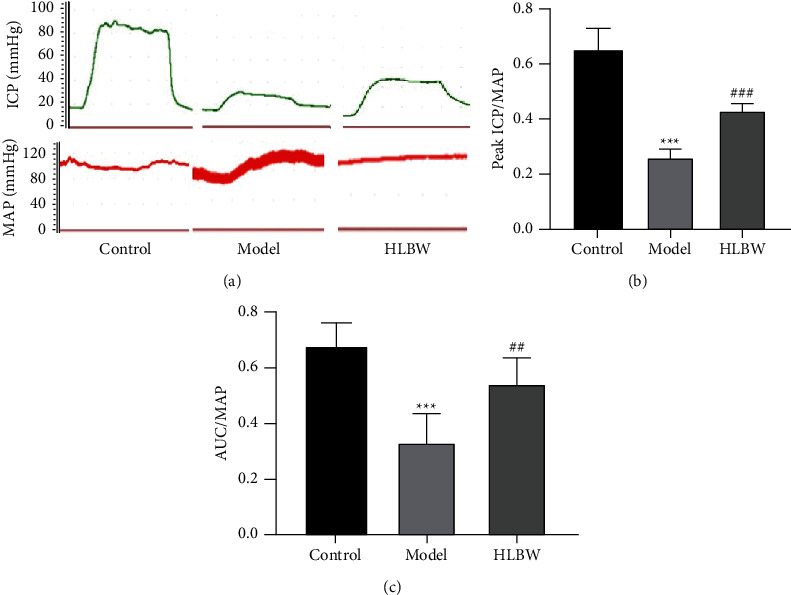
Effect of HLBW on the erectile function of DMED rats. (a) Representative traces of ICP and MAP with stimulation at 5.0 V for 1 min. (b) The ratios of peak ICP and (c) the ratios of AUC to MAP in the three groups are presented through bar graphs. *Note.*^*∗∗∗*^*P* < 0.001 vs. control group; ^*###*^*P* < 0.001, ^*##*^*P* < 0.01 vs. model group. Data are expressed as the mean ± SD. ICP: intracavernous pressure; AUC: area under the curve.

To evaluate the effect of HLBW on erectile function, the changes in ICP and MAP induced by stimulating the cavernous nerve are shown in Figure 2(a). The ratios of maximal ICP/MAP and AUC/MAP are shown in Figures 2(b) and 2(c). There were lower ratios of maximal ICP/MAP and AUC/MAP in the DMED group than in the nondiabetic control group (*P* < 0.001). HLBW treatment significantly increased the ratios compared with those in the DMED group (*P* < 0.001, *P* < 0.01).

### 3.4. HLBW Treatment Suppresses Oxidative Stress in the CC of DMED Rats

**Figure 3:**
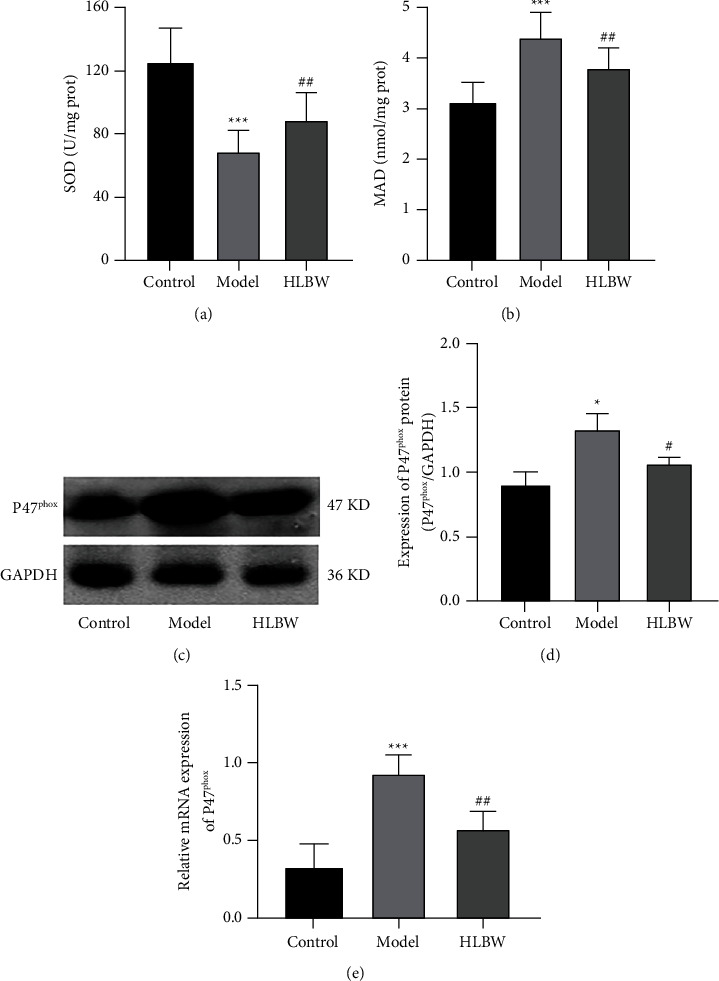
Effect of HLBW on oxidative stress in the CC of DMED rats. (a) SOD activity and (b) MDA level in the CC of the three groups. (c) Western blot analysis of P47^phox^ protein expression. (d) Relative expression levels of P47^phox^ normalized to GAPDH expression. (e) RT-PCR for the mRNA expression of P47^phox^. *Note.*^*∗∗∗*^*P* < 0.001, ^*∗*^*P* < 0.05 vs. control group; ^##^*P* < 0.01, ^#^*P* < 0.05 vs. model group. Data are expressed as the mean ± SD. SOD: superoxide dismutase; MDA: malondialdehyde.

ROS are regarded as critical factors in the progression of DM complications, and the NADPH oxidase subunit P47^phox^ is a crucial resource of ROS. To demonstrate the role of HLBW in oxidative stress, we detected the levels of SOD and MDA, as well as the expression level of NADPH oxidase subunits P47^phox^, among the three groups. As shown in Figures 3(c)–3(e), the mRNA and protein expression levels of p47^phox^ were higher in the DMED group than in the normal control group (*P* < 0.001, *P* < 0.05), while HLBW treatment decreased the expression levels of p47^phox^ (*P* < 0.01, *P* < 0.05). Consistent with p47^phox^ expression, the DMED group also exhibited lower SOD activity and higher MDA levels than the control group (*P* < 0.001). After HLBW treatment, SOD activity was obviously elevated and MDA levels were markedly decreased compared with those in the DMED group (*P* < 0.01) in Figures 3(a) and 3(b).

### 3.5. HLBW Treatment Inhibited Apoptosis in the CC of DMED Rats

**Figure 4:**
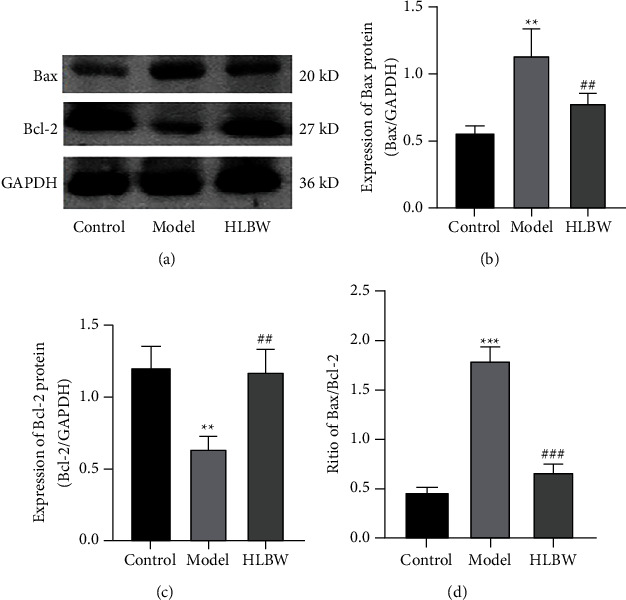
Effect of HLBW on apoptosis in the CC of DMED rats. (a) Western blot analysis of Bax and Bcl-2 protein expression. (b, c) Relative expression levels of Bax and Bcl-2 normalized to GAPDH expression. (d) The ratio of Bax/Bcl-2 in different groups. *Note.*^*∗∗∗*^*P* < 0.001, ^*∗∗*^*P* < 0.01 vs. control group; ^###^*P* < 0.001, ^##^*P* < 0.01 vs. model group. Data are expressed as the mean ± SD.

To evaluate the apoptosis degree of the corpus cavernosum among the three groups, we detected the expression of proapoptotic factors (Bax) and antiapoptotic factors (Bcl-2) through Western blot analysis. As shown in Figure 4, in the DMED group, the protein expression of Bax was significantly higher, while the expression of Bcl-2 was obviously lower, than that in the control group (*P* < 0.01). HLBW treatment decreased the protein expression of Bax and suppressed the expression of Bcl-2 (*P* < 0.01). The ratio of Bax/Bcl-2 in the DMED group was obviously higher than that in the control group (*P* < 0.001), while the ratio was decreased in the HLBW group compared with the DMED group (*P* < 0.001). The results suggested that HLBW treatment could alleviate the apoptosis of corpus cavernosum in DMED rats.

### 3.6. HLBW Treatment Restored the Endothelial Function in the CC of DMED Rats

**Figure 5:**
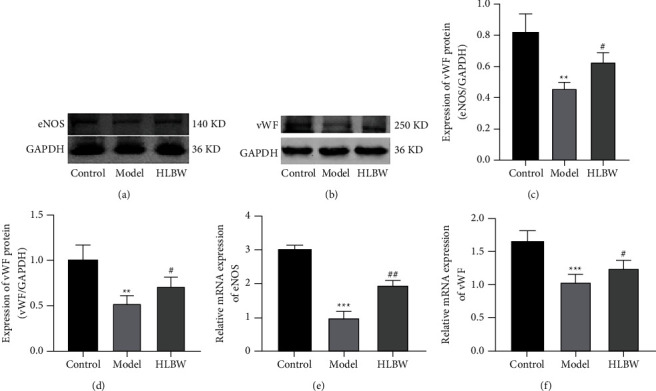
Effect of HLBW on endothelial function in the CC of DMED rats. (a, b) Western blot analysis of eNOS and vWF protein expression. (c, d) Relative expression levels of eNOS and vWF normalized to GAPDH expression. (e, f) RT-PCR for the mRNA expression of eNOS and vWF. *Note.*^*∗∗∗*^*P* < 0.001, ^*∗∗*^*P* < 0.01 vs. control group; ^##^*P* < 0.01, ^#^*P* < 0.05 vs. model group. Data are expressed as the mean ± SD. eNOS: endothelial nitric oxide synthase; vWF: vascular endothelial marker.

Endothelial nitric oxide synthase (eNOS) is produced by endothelial cells of the corpus cavernosum, so the content of eNOS can reflect endothelial function to some extent. As illustrated in Figures 5(a), 5(c), and 5(e), the mRNA and protein expression levels of eNOS were significantly lower in the DMED group than in the nondiabetic control group (*P* < 0.001, *P* < 0.01). After HLBW treatment, the mRNA and protein expression levels of eNOS were elevated compared with those in the DMED group (*P* < 0.01, *P* < 0.05). The vascular endothelial marker (vWF) was utilized to indicate the content of the endothelium. As shown in Figures 5(b), 5(d), and 5(f), the mRNA and protein expression levels of vWF were reduced in the DMED group compared with those in the control group (*P* < 0.001, *P* < 0.01). After HLBW treatment, the mRNA and protein expression levels of vWF were higher than those in the DMED group (*P* < 0.05).

### 3.7. HLBW Treatment Improved the Smooth Muscle Content in the CC of DMED Rats

**Figure 6:**
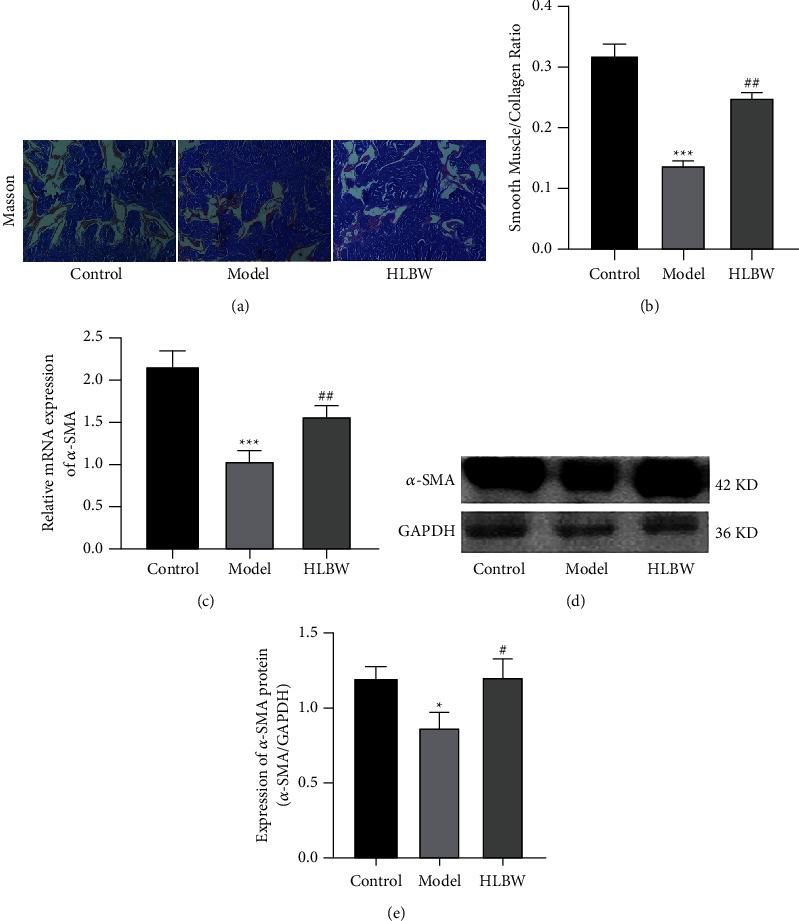
Effect of HLBW on smooth muscle content in the CC of DMED rats. (a) Masson trichrome staining (red indicates the area of smooth muscle, and blue indicates the area of collagen). (b) The ratio of smooth muscle to collagen in Masson-stained CC. (c) RT-PCR for the mRNA expression of *α*-SMA. (d) Western blot analysis for *α*-SMA protein expression. (e) Relative expression levels of *α*-SMA normalized to GAPDH expression. *Note.*^*∗∗∗*^*P* < 0.001, ^*∗*^*P* < 0.05 vs. control group; ^##^*P* < 0.01, ^#^*P* < 0.05 vs. model group. Data are expressed as the mean ± SD. *α*-SMA: *α*-smooth muscle actin.

Masson's trichrome staining was used to assess the content of smooth muscle in CC. As shown in Figure 6(a), the red staining areas represent smooth muscle and the blue staining areas represent collagen fiber deposition. In the control group, the structure of the red region was intact, the blood sinuses were abundant, and smooth muscle fibers could be seen between collagen fibers. While we observed that the structure of smooth muscle was broken, the number of blood sinuses was decreased, and smooth muscle fibers between collagen fibers were rare in the DMED group. HLBW treatment improved the structure of smooth muscle and increased the content of smooth muscle. As shown in Figure 6(b), the ratio of smooth muscle to collagen was obviously lower in the DMED group than in the control group (*P* < 0.001), while the ratio was higher in the HLBW group than in the DMED group (*P* < 0.01). *α*-Smooth muscle actin (*α*-SMA) was used to indicate the content of smooth muscle. As shown in Figures 6(c)–6(e), the mRNA and protein expression levels of *α*-SMA were lower in the DMED group than in the control group (*P* < 0.001, *P* < 0.05). After HLBW treatment, the mRNA and protein expression levels of *α*-SMA were markedly higher than those in the DMED group (*P* < 0.01, *P* < 0.05).

## 4. Discussion

DM is a complex metabolic syndrome characterized by hyperglycemia that leads to vascular complications. ED is a common complication of diabetes and is considered an early warning sign for the development of vascular disease. DM can destroy the vascular endothelium of the penis, resulting in an inability to achieve and maintain an erection of the penis [20]. In this study, we established a model of diabetic rats through injection of STZ and then screened DMED rats by an APO test. DMED rats were treated with HLBW for 12 weeks. It was very exciting to find that HLBW treatment markedly increased the ratio of ICP/MAP in DMED rats, indicating that HLBW can improve erectile dysfunction in diabetic rats. At the same time, our experimental results showed that HLBW lowered glucose levels and increased insulin sensitivity compared with those in the model group, which suggested that HLBW has potential therapeutic value for diabetes.

HLBW is a classical herbal prescription composed of TFG and PC. Based on the HPLC results in our study, psoralenoside, isopsoralenoside, rutin, quercitrin, psoralen, isopsoralen, and bakuchiol were well identified in HLBW granules. Several studies have revealed that rutin can lower plasma glucose levels and ameliorate hyperglycemia-induced oxidative damage in diabetic rats [21, 22]. A recent study showed that quercitrin could control diabetes and ameliorate liver damage in STZ-induced diabetic rats [23]. A previous study demonstrated that the major components of PC, psoralen and isopsoralen, could protect pancreatic beta cells from oxidative stress damage caused by diabetes [15]. In summary, these chemical components of HLBW have hypoglycemic and antioxidative activities. Our experiment explored the possible mechanism of HLBW in treating erectile dysfunction due to diabetes.

Prolonged hyperglycemia leads to the production of reactive oxygen species (ROS). Numerous studies have shown that oxidative stress could decrease the blood flow of the cavernous sinus, interfere with endothelial function, and impair muscle relaxation in the corpus cavernosum, which plays an important role in the occurrence and development of diabetic erectile dysfunction [20, 24]. MDA is a terminal product of lipid oxidation, the content of which reflects the degree of oxidative stress damage. SOD is a major enzyme that can remove excessive ROS and is considered as a marker of the body's antioxidant abilities. To investigate the effect of HLBW on oxidative stress in CC, we examined the level of MDA and the activity of SOD in the CC of the three groups. Consistent with our previous study [18], we observed a higher MDA level and a lower SOD activity in the model group; however, after HLBW intervention, the MDA level significantly decreased and the SOD activity obviously increased, indicating that HLBW could suppress oxidative stress in the CC of DMED rats. Some studies have demonstrated that NADPH oxidase is a major source of ROS in the development of impaired endothelium-dependent vasorelaxation [25]. The expression of NADPH oxidase subunits, such as p47phox and p67phox, was demonstrated to be elevated in the penis of diabetic erectile dysfunction rats [26]. In accordance with previous experiments, our present study showed that the mRNA and protein expression levels of p47^phox^ were higher in the CC of the DMED group than in normal rats. However, HLBW treatment decreased the mRNA and protein expression of p47^phox^ in the CC of DMED rats. The results indicated that HLBW could reduce oxidative stress caused by inhibiting NADPH oxidase in the CC of DMED rats.

Oxidative stress can induce CC cell apoptosis in an environment of prolonged hyperglycemia, leading to erectile dysfunction [27, 28]. Our data verified that HLBW intervention could inhibit CC cell apoptosis by detecting the expression of proapoptotic factors (Bax) and antiapoptotic factors (Bcl-2). In our study, we found that the expression of Bax was elevated and the expression of Bcl-2 was decreased in DMED rats, suggesting that oxidative stress could disrupt the balance between Bax and Bcl-2. Meanwhile, the ratio of Bax/Bcl-2 was significantly higher in DMED rats than in normal rats. After HLBW treatment, the protein expression of Bax was decreased, the expression of Bcl-2 was increased, and the ratio of Bax/Bcl-2 was lower in DMED rats.

The endothelium and smooth muscle cells in the CC play an important role in penile erection [29]. Endothelial cells of CC are conducive to the production of nitric oxide (NO), which can stimulate the formation of cyclic guanosine monophosphate (cGMP). Upregulation of the NO/cGMP signaling pathway leads to smooth muscle relaxation and can ultimately contribute to facilitating penile erection [30]. It has been proven that DM-induced oxidative stress can promote eNOS dysfunction by uncoupling and reducing the formation of NO, which impairs the endothelial function of the CC in the penis [31]. Our research demonstrated that the mRNA and protein expression levels of eNOS were lower in the DMED group, while HLBW treatment increased eNOS expression in the CC. Meanwhile, we found that the mRNA and protein expression levels of vascular endothelial marker (vWF), which was used to indicate the content of endothelium, were significantly decreased in the CC of DMED rats, which is consistent with previous studies [32]. After HLBW treatment, the expression of vWF was increased compared with that in the DMED group.

Vascular smooth muscle marker (*α*-SMA) has been proven to be a remarkable molecular marker of smooth muscle cell phenotype [33]. Masson staining can dye different kinds of muscle fibers in different colors so that the relationship between the number and proportion of muscle fibers can be observed under a light microscope. The ratio of smooth muscle fiber to collagen fiber can reflect erectile function. Compared with the normal group, the expression of *α*-SMA was decreased and a lower ratio of smooth muscle to collagen was observed in DMED rats. However, HLBW treatment could restore pathological changes in smooth muscle cells. The results demonstrated that HLBW could have a potential protective role in the endothelium and smooth muscle cells of the CC.

## 5. Conclusions

In summary, our results demonstrated that HLBW reduced blood glucose and ameliorated insulin resistance. Meanwhile, HLBW protected the endothelium and smooth muscle cells of the CC by inhibiting oxidative stress and apoptosis and thereby improved the erectile function of DM rats. The therapeutic characteristics of HLBW, which combines lowering glucose and improving erectile dysfunction, provide ideas for the development of new drugs. We will further explore the specific mechanism of HLBW inhibiting oxidative stress in the CC.

## Figures and Tables

**Table 1:** Primer sequences used for real-time PCR.

Gene		Primer sequence (5′-3′)
P47^phox^	Forward	5′-TTCAGACCTATCGGGCCATC-3′
	Reverse	5′-CTCGGTTTGGCTTCATCTGG-3′

vWF	Forward	5′-TTGCTCAGGGACATGGCTTA-3′
	Reverse	5′-AGGTGAGGGCCAGAACTAAC-3′

*α*-SMA	Forward	5′-CATCATGCGTCTGGACTTGG-3′
	Reverse	5′-CCAGGGAAGAAGAGGAAGCA-3′

eNOS	Forward	5′-GATCCTAACTTGCCTTGCATCCT-3′
	Reverse	5′-TGTAATCGGTCTTGCCAGAATCC-3′

GAPDH	Forward	5′-GTGACACCCACTCTTCCACC-3′
	Reverse	5′-GTGGTCCAGGAGGCTCTTAC-3′

**Table 2:** Effect of HLBW on body weight and biochemistry parameters in DMED rats. ^*∗∗∗*^*P* < 0.001*P* < 0.01*P* < 0.05.

	Control	Model	HLBW
Weight (g)	480.1 ± 23.02	267.9 ± 20.41^*∗∗∗*^	319.6 ± 43^#^
FBG (mmol/L)	0.24 ± 0.13	26.5 ± 1.59^*∗∗∗*^	22.86 ± 3.05^##^
FINS (mU/L)	1.74 ± 0.13	5.20 ± 0.16^*∗∗∗*^	4.25 ± 0.18^##^
HOMA-IR	0.41 ± 0.03	6.12 ± 0.38^*∗∗∗*^	4.31 ± 0.50^##^

Note:^*∗∗∗*^*P* < 0.001 vs. control group; ^*##*^*P* < 0.01, ^*#*^*P* < 0.05 vs. model group. The data are shown as the means ± SD.

## Data Availability

The datasets used and analyzed during the current study are available from the corresponding author upon request.

## Abbreviations

HLBW: Hu-Lu-Ba-Wan
DMED: Diabetes mellitus-induced erectile dysfunction
TFG: *Trigonella foenum-graecum* L
PC: *Psoralea corylifolia* L
ICP: Intracavernous pressure
MAP: Mean arterial pressure
*α*-SMA: *α*-Smooth muscle actin
eNOS: Endothelial nitric oxide synthase
NADPH: Nicotinamide adenine dinucleotide phosphate
vWF: Vascular endothelial marker
DM: Diabetes mellitus
ED: Erectile dysfunction
ROS: Reactive oxygen species
SOD: Superoxide dismutase
MDA: Malondialdehyde
HPLC: High-performance liquid chromatography
FBG: Fasting blood glucose
FINS: Fasting serum insulin
IR: Insulin resistance
CC: Corpus cavernosum.
